# E-CHARM Heart Age Trial in Primary Care: Design and Baseline Findings

**DOI:** 10.1093/geroni/igaf122.3575

**Published:** 2025-12-31

**Authors:** Guanzhou Wang, Lei Wu, Weijing Song, Yuzhou Sun, You Zuo, Yuxuan Huang, Boyuan Li

**Affiliations:** Duke University, Durham, North Carolina, United States; Duke Kunshan University, Kunshan, Jiangsu, China (People’s Republic); Duke Kunshan University, Durham, North Carolina, United States; University of St. Andrews, St. Andrews, Scotland, United Kingdom; Duke Kunshan University, Kunshan, Jiangsu, China (People’s Republic); Duke Kunshan University, Kunshan, Jiangsu, China (People’s Republic); Duke Kunshan University, Kunshan, Jiangsu, China (People’s Republic)

## Abstract

Cardiovascular risk awareness among older adults often fails to align with physiological risk, limiting prevention. “Heart Age” Re-frames risk as an intuitive biological age; however, evidence from community primary care, particularly in resource-limited settings, remains scarce. We designed an individually randomized trial in a community health system to test a structured Heart Age risk-education report generated from ASCVD components fully extracted from Chinese government-funded annual physical exams and delivered with a brief physician explanation and tailored lifestyle guidance during routine follow-up, with no extra tests or visits. Older adults with hypertension were allocated to usual care plus the Heart Age report (intervention) or usual care (control). Baseline measures included demographics, a 10-item cardiovascular risk awareness score (0–10), lifestyle indicators (e.g., fruit-intake days/week), medication adherence by the 4-item Morisky scale (MMAS-4; 0–4, higher=better), and routine clinical data. We enrolled 378 participants (174 intervention; 204 control), mean age 66.8±12.0 years. The awareness score averaged 7.16/10; fruit-intake frequency was median 5 days/week (IQR 3–7); medication adherence was mean 2.71/4, median 3 (IQR 2–4). Among intervention participants with complete Heart Age data (n = 174), the Heart Age gap (Heart Age − chronological age) was 8.2±11.7 years. Risk awareness showed near-zero correlation with the Heart Age gap (r≈0.05, 95% CI − 0.10 to 0.20); age-sex adjustment gave similar results. Leveraging routinely collected exams to produce an ASCVD-based Heart Age report with brief lifestyle counseling appears feasible at no added cost; the baseline misalignment highlights room, and opportunity, for improved risk communication. Endline effectiveness evaluation is ongoing.

